# Plasmodium falciparum, Vivax and Scrub Typhus in a Young Adult Male: Surviving a Triple Strike

**DOI:** 10.7759/cureus.30176

**Published:** 2022-10-11

**Authors:** Vijay Kota, Ruchita Kabra, Sunil Kumar, Sourya Acharya, Rinkle R Gemnani

**Affiliations:** 1 Medicine, Jawaharlal Nehru Medical College, Wardha, IND; 2 Internal Medicine, Jawaharlal Nehru Medical College, Wardha, IND

**Keywords:** zoonotic, co-infection, malaria, rainy, plasmodium

## Abstract

Malaria is caused by Plasmodium species and is called a “Rainy season disease”. Scrub typhus is a zoonotic disease caused by Orientia tsutsugamushi. Both diseases occurring as co-infection are very rare. If a patient presents with a fever complaint, all the possible causes should be ruled out even if the patient shows infectivity for one of the vector-borne diseases. This case report shows the rare occurrence of the co-infection and its presentation. Early detection and timely management can prevent the patient from various fatal complications.

## Introduction

Malaria is a severe parasite illness that causes significant morbidity and mortality in India. Early diagnosis and comprehensive therapy are crucial to controlling the disease [1]. The Indian government's national vector-borne illness management program reports about 1.5 million confirmed cases annually. *P. falciparum* is responsible for nearly half of these cases. Malaria can be cured if therapy is begun early enough. Treatment delays might have catastrophic effects, including death. Controlling disease transmission also necessitates prompt and efficient treatment. Patients who live in or have recently visited endemic regions should be suspected of having malaria. The incubation period of *P. falciparum* malaria is 12-14 days, and plasmodium vivax is 12-17 days [2]. Scrub typhus is an uncommon rickettsial illness found in Himachal Pradesh. It is caused by the bacterium *orientia tsutsugamushi*, which is spread by trombiculid mite larvae. Eschar may or may not be visible in every situation [3,4]. Though it is uncommon in the plains, it should be included in the differential for a patient with a fever and seizure.

## Case presentation

**Figure 1:**
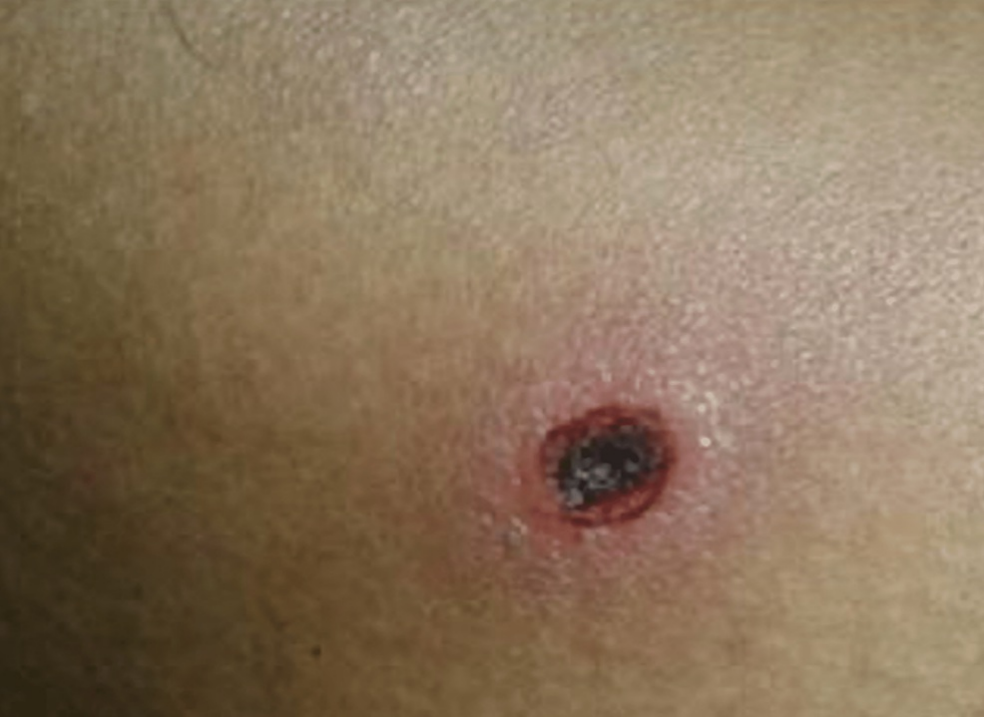
Blackish eschar on the back showing portal for Scrub typhus.

A 24-year-old male came to the hospital with complaints of low-grade fever for eight days, irritability, and disoriented for one day. A blackish rash on the back of the chest, as shown in Figure 1. No history of cough, cold, nausea, vomiting, abdominal pain, breathlessness, loss of consciousness, seizures, chest pain, or palpitations. The patient’s general condition was poor, afebrile, blood pressure 90/70 mm Hg, pulse rate 102/min,spo2 98% on room air, and central nervous system examination revealed that the patient was conscious and disoriented. Another systemic examination was within normal limits.

**Figure 2:**
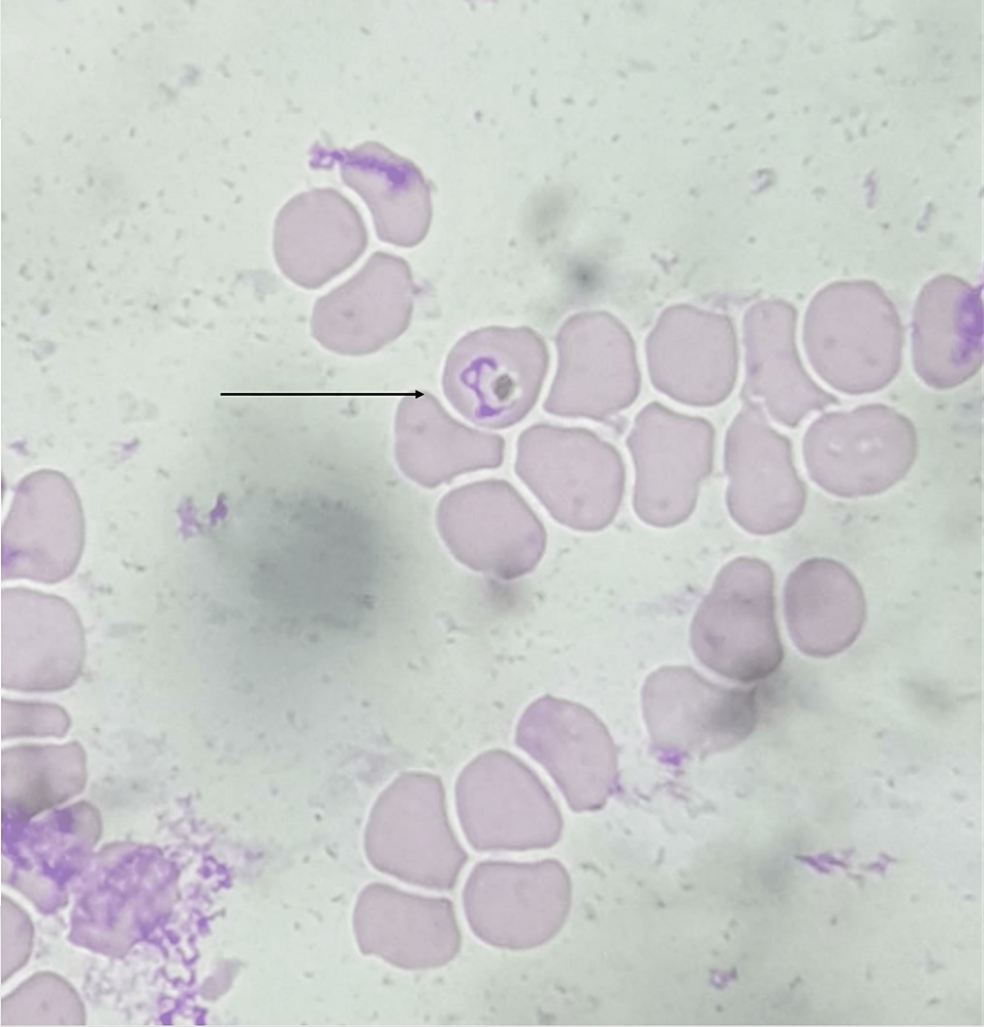
Peripheral smear showing haemoparasite with ring of plasmodium vivax , plasmodium falciparum.

**Figure 3:**
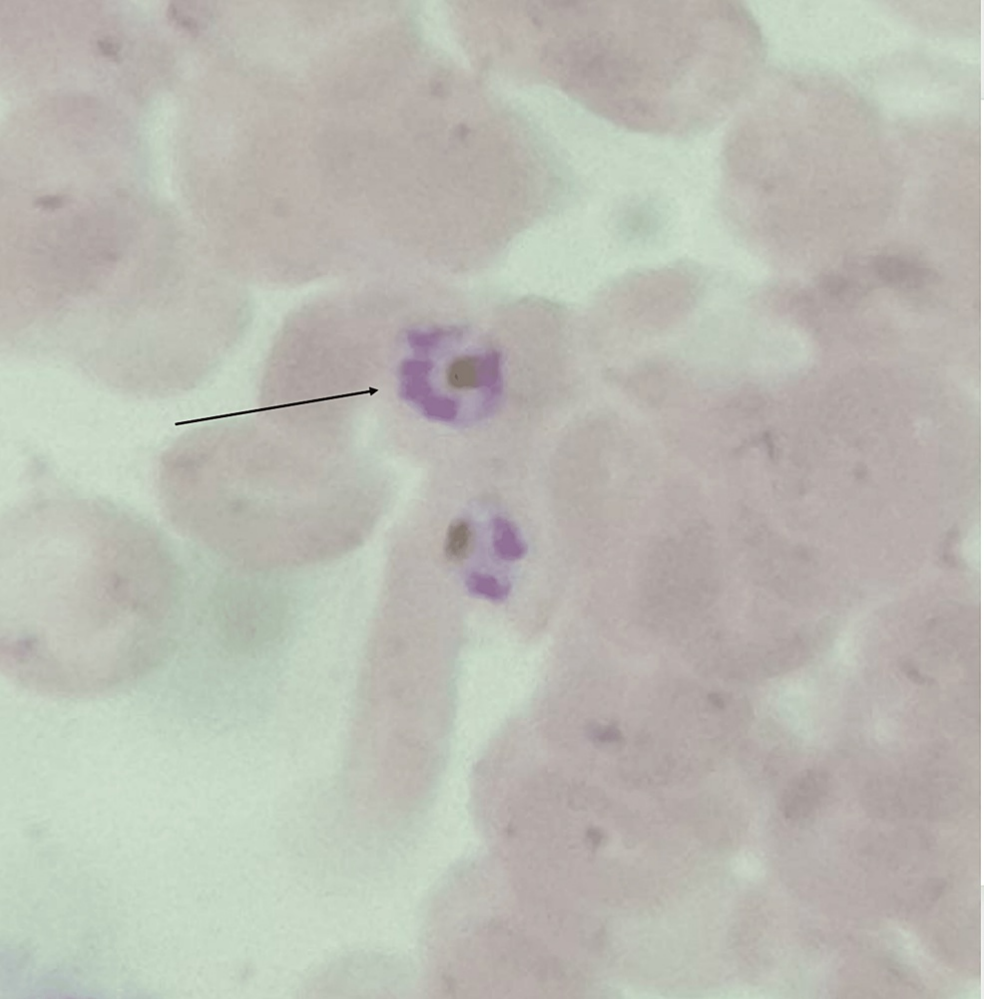
Peripheral smear showing schizonts and gametocytes of plasmodium vivax.

Investigations were done as shown in Table 1, and peripheral smear suggestive of haemoparasite -ring of plasmodium vivax, plasmodium falciparum as shown in Figure 2 with schizonts and gametocytes of plasmodium vivax as shown in Figure 3 impression of peripheral smear positive for malarial parasites with moderate thrombocytopenia with mild hypochromic anemia. Cerebrospinal fluid (CSF) analysis was normal.

**Table 1:** Laboratory investigations at the time of admission SGOT (Serum glutamic-oxaloacetic transaminase), SGPT (Serum glutamic-pyruvic transaminase), APTT (Activated Partial Thromboplastin Time), PT (Prothrombin time), INR  (International Normalised Ratio), CRP (C-reactive protein)

Investigations	Patient’s value	Normal range
Hemoglobin	6.4 gm%	12-14gm%
White blood cell count	8500 cu/mm	6000-11000 cu/mm
Platelet count	17,000 lakh/mm	1.5-4.5 lakh/mm
APTT	30.2 sec	29.5
PT	14.2 sec	11.9
INR	1.20 sec	<1.4
CRP	24 mg/dl	<1 mg/dl
Urea	70 mg/dl	9-20 mg/dl
Creatinine	1.1 mg/dl	0.6-1.2mg/dl
Sodium	143 mmol/L	135-145 mmol/L
Potassium	5.3 mmol/L	3.5-5.5 mmol/L
Alkaline phosphatase	105 U/L	38-126 U/L
SGOT	54 U/L	<50 U/L
SGPT	28 U/L	17-59 U/L
Total Bilirubin	5.4 mg/dl	0.2-1.3mg/dl
Unconjugated Bilirubin	3.2 mg/dl	<0.3 mg/dl
Conjugated bilirubin	2.2 mg/dl	<1.1 mg/dl

The patient was initially started with injectable Artesunate 120 mg at 0-12hour-24hour followed by 120 mg for three days, injectable doxycycline 100 mg twice a day, tablet primaquine 15 mg twice a day and other supportive medications. The patient's condition improved during the hospital stay and symptomatically better. After completing an intravenous anti-parasitic drugs for seven days, the patient was discharged. On discharge, patient was adviced for screening of the relatives. On follow-up, the patient was doing well. 

## Discussion

India is a tropical nation where *Plasmodium vivax (P. vivax) *and *Plasmodium falciparum (P. falciparum) *malaria are prevalent. Despite the fact that malaria has symptoms that are similar to those of many other infectious illnesses, all clinically suspected malaria cases should be explored using microscopy and/or a Rapid Diagnostic Test (RDT) as soon as possible [5]. The RDT employed, in this case, was the advantage mal card, which is a J. Mitra and Co. Pvt. Ltd. immunoassay based on the "sandwich" concept. The kit has a 100 percent sensitivity for *P. vivax* and a 95.83 percent malaria-negative specificity. To confirm the co-infection by *P. vivax* and *P. falciparum*, a thick and thin film analysis of the peripheral smear was performed [6]. The patient's financial situation prevented him from undergoing the Polymerase Chain Reaction (PCR), which is a costly operation. PCR-based diagnostic techniques enable Plasmodium species identification in clinical settings with adequate apparatus, even when parasitemias are below blood smear sensitivity limits. These more sensitive tests' results are frequently beneficial in formulating treatment decisions for species (*P. vivax* and *P. ovale*) capable of generating latent liver stages and eventual malarial relapses [7]. PCR, despite its benefits, is unlikely to be helpful outside of well-equipped laboratories with a steady supply of energy and costly equipment. The goal of early identification and treatment of malaria patients is to achieve a full cure, avoid the development of uncomplicated malaria to severe illness, prevent fatalities, stop transmission, and reduce the danger of drug-resistant parasite selection and dissemination [8].

Scrub typhus should be suspected in every instance with protracted fever, and a thorough search for eschar should be conducted, as eschar is the most helpful clinical diagnostic clue [1]. *Plasmodium vivax* can remain latent in liver cells as hypnozoites, which can evolve into merozoites months or years later. Scrub typhus has a nine-day incubation period. As a result, the patient who was already suffering from *Plasmodium vivax* fever might have been infected with scrub typhus [3]. Scrub typhus is usually milder in those who live in endemic areas, and there is seldom any rash or eschar. Scrub typhus has been recorded in rising numbers across India, particularly in the Himalayan foothills, Assam, West Bengal, and Tamil Nadu. Scrub typhus has resurfaced in numerous places in India, particularly South India, according to recent reports [4]. Scrub typhus is severely underdiagnosed in India due to its non-specific clinical presentation, doctors' lack of awareness and suspicion, and lack of diagnostic resources [7]. Empirical therapy with doxycycline or macrolides may be provided in situations where there is a high suspicion of scrub typhus since delay in treatment can result in serious life-threatening consequences [4].

## Conclusions

Malarial and scrub typhus infection can be controlled with different measures taken at different levels. Early diagnosis and prompt treatment can help in preventing life-threatening complications. Along with treatment, and prevention at different levels vector control measures to public health education for awareness of transmission, symptoms, and prevention of diseases is important, especially in endemic areas. 
